# Efficacy and safety of anlotinib plus XELOX regimen as first-line therapy for mCRC: a single-arm, multicenter, phase II study (ALTER-C-001)

**DOI:** 10.3389/fonc.2023.1238553

**Published:** 2023-09-01

**Authors:** Bo Song, Hai Hu, Li Zhang, Su-Juan Ye, Yong-Dong Jin, Chang-Ling Shang, Jun Zhang, Hao Sun, Ke Zhang, Bo Yi, Yun-Wei Han, Jin Yan

**Affiliations:** ^1^ Department of Gastrointestinal Surgery, Sichuan Cancer Hospital and Institute, Sichuan Cancer Center, School of Medicine, University of Electronic Science and Technology of China, Chengdu, China; ^2^ Department of Medical Oncology, Chongqing University Three Gorges Hospital, Chongqing, China; ^3^ Department of Medical Oncology, The Affiliated Hospital of Southwest Medical University, Luzhou, China; ^4^ Department of Medical Oncology, Sichuan Cancer Hospital and Institute, Sichuan Cancer Center, School of Medicine, University of Electronic Science and Technology of China, Chengdu, China; ^5^ Gastrointestinal Cancer Center, Chongqing University Cancer Hospital, Chongqing, China

**Keywords:** anlotinib, XELOX, metastatic colorectal cancer, efficacy, safety, first-line therapy

## Abstract

**Background:**

Anlotinib showed encouraging anti-tumor activity in metastatic colorectal cancer (mCRC). This study was designed to assess the efficacy and safety of anlotinib plus XELOX as first-line therapy in mCRC patients.

**Materials and Methods:**

Eligible patients aged ≥18 with mCRC were enrolled in this multicenter, single-arm, phase II, exploratory study. Patients received at least 6 cycles of anlotinib, oxaliplatin, and capecitabine as initial therapy. Subsequently, patients received anlotinib monotherapy as maintenance therapy until tumor progression or intolerable toxicity. The primary endpoint was progression-free survival (PFS).

**Results:**

Thirty-one patients were included between December 2019 and March 2022. The median follow-up was 17.5 (95% CI, 3.0-17.5) months. The median PFS was 8.3 (95% CI, 6.3-10.0) months, with 6- and 12-month PFS rates of 82.3% (95% CI, 59.2%-93.0%) and 18.9% (95% CI, 4.8%-40.1%), respectively. Fifteen (48.4%) achieved partial response for an ORR of 48.4% (95% CI, 30.2%-66.9%). The disease control rate was 71.0% (95% CI, 52.0%-85.8%) due to 7 (22.6%) stable diseases. The median duration of response was 6.0 (95% CI, 3.6-8.0) months and 1 patient had the longest ongoing response of 17.3 months. Of 24 patients with evaluable imaging, 23 (74.2%) obtained tumor shrinkage. The median PFS (11.0 vs. 6.9 months) and ORR (66.7% vs. 60.0%) for patients with *RAS/BRAF* wild-type were numerically better than those with mutation. Three patients are still ongoing treatment. The grade 3 or more treatment-emergent adverse events (TEAEs) were mainly hypertension (12.9%) and decreased neutrophil count (12.9%). Four (12.9%) had serious TEAEs, primarily including abdominal pain and incomplete intestinal obstruction.

**Conclusion:**

Anlotinib plus XELOX as first-line therapy in patients with mCRC showed anti-tumor activity and safety profile, which is worth further investigation.

**Clinical Trial Registration:**

chictr.org.cn, identifier ChiCTR1900028417.

## Introduction

Colorectal cancer (CRC) has become the third most lethal cancer worldwide with almost 900, 000 deaths annually (1). Among them, 20% of patients have developed metastases at diagnosis (1). As the disease progressed, up to 40% of CRC patients had metastatic disease (2). Moreover, the prognosis with a 5-year survival rate of <20% in patients with metastatic CRC (mCRC) is far from satisfactory (3). Therefore, it is necessary to develop effective therapy with improved survival.

The primary treatment of unresectable mCRC is systemic therapy, including cytotoxic chemotherapy and biological therapy (1). Effective first-line therapy is a key determinant of successful systemic therapy for the majority of mCRC patients (4). Compared with the first-line therapy, subsequent therapies address only a subset of patients, who may present with reduced tolerance of toxicity, resulting in short treatment durations (5). Since angiogenesis is a hallmark of cancer (6), accumulating evidence reported the encouraging efficacy of anti-angiogenesis plus chemotherapy in the first-line setting for mCRC (7). Especially, the landmark AVF2107 trial demonstrated that bevacizumab plus chemotherapy significantly improved progression-free survival (PFS: 10.6 vs. 6.2 months; hazard ratio [HR]: 0.54) and overall survival (OS: 20.3 vs. 15.6 months; HR: 0.66) in patients with mCRC (8). Although anti-angiogenesis has become an appealing first-line choice for mCRC patients, few drugs were proven to be effective other than bevacizumab (7). Thus, additional investigations of combination regimens involving more promising anti-angiogenesis agents and chemotherapy as the first-line therapy remain necessary.

The expressions of VEGF, VEGF receptor 1/2, and molecules involved in proangiogenic pathways, such as fibroblast growth factor and platelet-derived growth factor, have been detected in CRC patients (9, 10). However, small-molecule tyrosine kinase inhibitor (TKI) was just the third-line therapy for mCRC when this phase II study was designed in 2019, and there were only two phase II studies on the efficacy evaluation of TKI in the first-line setting (11, 12). In parallel, there was a lack of accessibility to cetuximab due to its high cost in China in 2019, and only bevacizumab was frequently used in Chinese clinical practice; further, optimal targeted therapy remained undefined for mCRC in 2019 in China. Thus, we were eager to provide a new first-line targeted combination therapy for Chinese mCRC patients.

Anlotinib, as a novel oral small-molecule TKI with multi-target (13, 14), has shown encouraging anti-tumor effects and manageable safety in various solid tumors (14, 15). Thrillingly, the randomized phase III trial has demonstrated that mCRC patients without remission following standard therapy achieved impressive improvements in PFS, objective response rate (ORR), and disease control rate (DCR) in the anlotinib group over the placebo group (16). Furthermore, previous studies also revealed that anlotinib plus chemotherapy was a promising second-line therapy with an encouraging efficacy and safety profile for mCRC (17). In parallel, anlotinib-based regimens have been applied as the first-line therapy in other tumors and exhibited favorable anti-tumor activity and safety (18, 19).

In our study, XELOX was considered one of the ideal chemotherapy options based on previous studies (20). Taken together, we conducted a phase II study in 2019 to assess the efficacy and safety of the combination with first-line anlotinib plus XELOX in patients with mCRC.

## Materials and methods

### Study design

The ALTER-C-001 study was a multicenter, single-arm, phase II, exploratory study (Study registration ID: ChiCTR1900028417) and was conducted at 4 centers in Sichuan and Chongqing, China. Each center had independent ethics committee that approved the research protocol. The primary goal of this study was to evaluate the efficacy and safety of anlotinib plus XELOX in patients with mCRC. The study was performed in accordance with the principles of Good Clinical Practice and the Declaration of Helsinki, as well as all relevant regulations and laws in the applicable countries. All patients gave written informed consent before enrollment.

### Patient population

Eligible patients were aged between 18 and 75 with histologically or cytologically confirmed metastatic colon and rectum adenocarcinoma (TNM stage IV). Patients had at least one untreated and measurable lesion within 3 months per Response Evaluation Criteria in Solid Tumors (RECIST) version 1.1 (21). Patients who received study treatment at least 2 weeks after palliative treatment in non-target lesions, or who received neoadjuvant/adjuvant chemotherapy, and targeted radio-/chemo-therapy for locally advanced treatment and relapsed more than 6 months from the last administration of peri-operation chemotherapy were included. Other inclusion criteria were Eastern Cooperative Oncology Group performance status (ECOG PS) of 0 or 1, adequate organ function, a minimum of six-month predicted survival duration, and left ventricular ejection fraction (LVEF) of ≥50%.

Patients who were diagnosed with mucinous adenocarcinoma or ovarian implantation metastasis and received prior antiangiogenic agents were ineligible. Patients who developed previous unrelieved treatment-related toxicity (≥grade 1) per the National Cancer Institute Evaluation Criteria for Common Adverse Events (NCI CTCAE), version 4.03 (22) were excluded from the study. Other main exclusion criteria included uncontrolled severe diseases, a history of psychotropic substance abuse with the inability to quit, and arterial and venous thrombosis within 6 months prior to the study. Pregnant or lactating patients, as well as patients with childbearing potential who did not use contraception if sexually active, were also excluded. Detailed exclusion criteria were listed in **Additional File 1: Table S1**.

### Procedures

**Figure 1:**
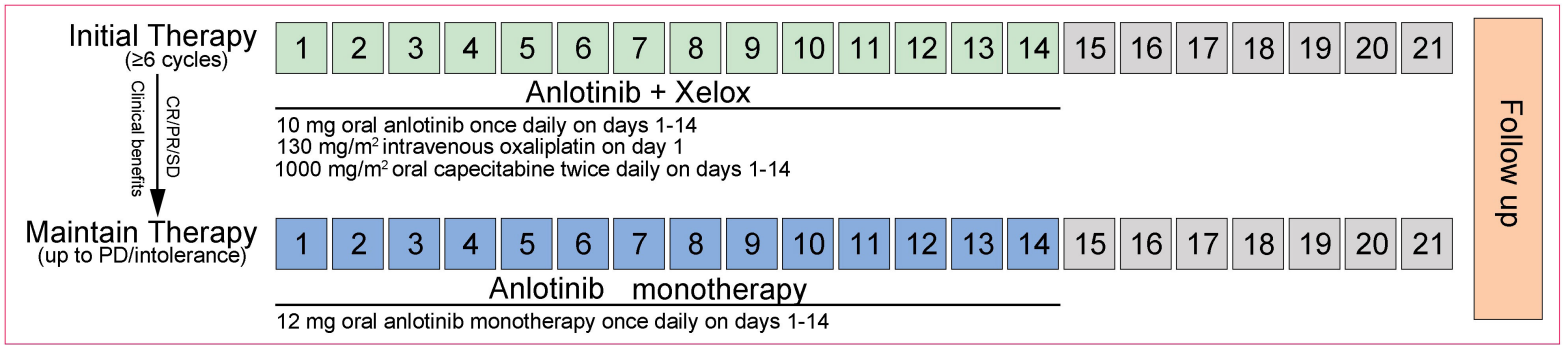
Treatment schedule (21-day cycle). The initial and maintenance therapy procedures for patients with mCRC. XELOX, oxaliplatin and capecitabine; CR, complete response; PR, partial response; PD, progressive disease; SD, stable disease.

Eligible patients received 10 mg oral anlotinib once daily on days 1 to 14, and the XELOX regimen (130 mg/m^2^ intravenous [>2 h] oxaliplatin on day 1, followed by 1000 mg/m^2^ oral capecitabine twice daily on days 1 to 14) as initial therapy on a 21-day cycle for a minimum of 6 cycles. Patients who had a complete response (CR)/partial response (PR), or stable disease (SD) in cycle 4 and cycle 6, or subsequent adjacent cycles during the initial therapy were then administered 12 mg oral anlotinib monotherapy as maintenance therapy once daily on days 1 to 14 every 3 weeks. In parallel, following the completion of 6 cycles of initial therapy, maintenance therapy in patients with achievements of CR/PR/SD while intolerance, or of clinical benefits with anlotinib alone per investigators was also recommended. The treatment was continued until progressive disease (PD), or intolerable toxicity. An overview of the therapeutic procedures was shown in **Figure 1**.

Dose modifications of any drug in this combination regimen were allowed depending on the potential therapeutic benefits for patients or treatment-emergent adverse events (TEAEs), which were determined by investigators. Dose reduction and interruption should give priority to anlotinib, then to chemotherapy. A maximum of two dose reductions was permitted (anlotinib: to 10 mg or even 8 mg; oxaliplatin: to 85 mg/m^2^ and then 65 mg/m^2^; capecitabine: to 75% or even 50% of the initial dose and then discontinuation). Patients could resume the initial dose or a reduced dose of anlotinib due to the recovery from toxicity. If the dose of capecitabine was reduced, it could not be increased in subsequent cycles. To allow a patient to recover from any toxicities, the drug could be interrupted. However, the cumulative duration of dose interruption should be limited to 2 weeks; otherwise, study treatment should be discontinued. Detailed criteria for dose modifications were listed in **Additional File 1: Table S2**.

### Assessments

Tumor response was assessed by investigators using magnetic resonance imaging (MRI) or computed tomography (CT) according to RECIST version 1.1 (21) at baseline and every 2 cycles until objective disease progression and intolerance. During the treatment, safety, and tolerability were monitored by physical examination, ECOG PS, vital signs, 12-lead electrocardiogram, and laboratory analyses (hematology, biochemistry, urinalysis, and coagulation). The TEAEs were evaluated by investigators and graded using the NCI-CTCAE, version 4.03 (22).

### Endpoints

The primary endpoint was PFS, which was defined as the time from the start of the treatment to disease progression (radiological or clinical progression) or death from any cause, whichever came first. The secondary endpoints were ORR (calculated as the proportion of patients achieving CR and PR), DCR (referred to the proportion of patients with CR, PR, and SD), duration of response (DoR, defined as the duration from the day when patients firstly had response [CR or PR] to the day that they had PD firstly or death from any cause), safety and tolerability. Safety and tolerability were assessed by TEAEs. A serious TEAE (SAE) was defined as any AE that was fatal, life-threatening, required prolonged hospitalization, and resulted in persistent or significant disability/incapacity.

### Statistical analysis

Sample size estimation was based on the primary endpoint of PFS. Approximately 13 PFS events were expected if 23 patients were enrolled within a 12-month accrual period with a 12-month follow-up. This number of events would provide 80% power at a two-sided 5% significance level, corresponding to the improvement in PFS from 6.6 months to 14 months based on the OPTIMOX-2 study (23). A total of 29 patients were required for this study, considering an approximate dropout incidence of 20%.

The efficacy analysis was performed in the full analysis set (FAS), which included all patients who received at least one dose of the study drug. Moreover, the analysis of ORR and DCR was additionally conducted in the per-protocol set (PPS; all FAS patients who had no protocol violations that directly impinged on or affected the efficacy endpoint and had evaluable imaging data). The safety analysis was performed in the safety analysis set (SAS), which was defined as all patients who received at least one dose of the study drug and had safety records.

Patient characteristics, safety outcomes, and tumor response were summarized descriptively. Categorical variables were summarized as frequencies (percentage [%]) and continuous variables were presented as medians with interquartile range (IQR) or range. The PFS and DOR were calculated by the Kaplan-Meier method with a 95% confidence interval (CI). The 95% CI of the ORR and DCR was calculated by the Clopper-Pearson method. Statistical comparisons of ORR according to *RAS/BRAF* status were performed using the Chi-square test or Fisher’s exact test, while those of PFS was performed using a two-sided exact log-rank test. All statistical tests were two-sided, with significance set at *p*<0.05. All analyses were conducted with obtained data, using SAS software version 9.4 (SAS Institute, Cary, NC, USA).

## Results

### Patient characteristics

**Figure 2:**
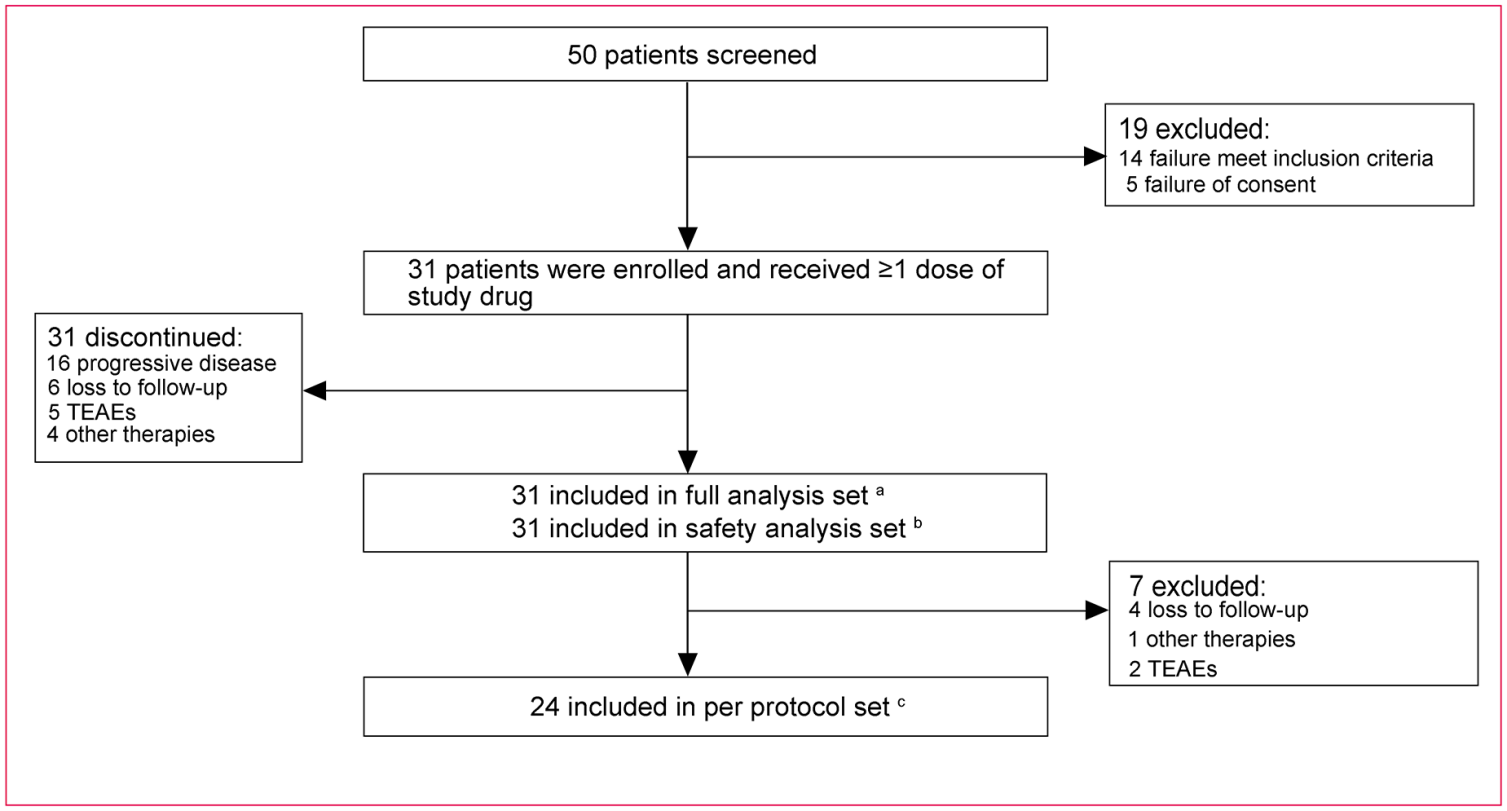
Study profile. ^a^ Thirty-one patients received at least one dose of the study drug. ^b^ Thirty-one patients received at least one dose of the study drug and had safety records. ^c^ Twenty-four patients received at least one dose of the study drug and meanwhile had no protocol violations that directly impinged on or affected the efficacy endpoint and had evaluable imaging data. XELOX, oxaliplatin and capecitabine; TEAEs, treatment-emergent adverse events.

Between December 2019 and March 2022, 50 mCRC patients from 4 centers across China were screened and 19 were excluded from enrollment due to failure to meet inclusion criteria (n=14) and failure of consent (n=5). A total of 31 patients were enrolled and received initial therapy and were included in the FAS and SAS (**Figure 2**). Moreover, 7 patients did not have available imaging data because of loss to follow-up (n=4), TEAEs (n=2), and other therapies (n=1); thus, 24 patients were included in the PPS.

The baseline characteristics of 31 patients were presented in **Table 1**. The median age of patients was 57 (range, 51-66) years. The majority (23/31, 74.2%) were male. The primary tumor sites were commonly found in the colon (13/31, 41.9%) and rectum (18/31, 58.1%). Most patients (27/31, 87.1%) had left colonic carcinoma. Nine (29.0%) mCRC patients were identified as *RAS/BRAF* wild-type; totally 10 (32.3%) cases did not detect mutation status due to financial burden. The majority (28/31, 90.3%) had ≥2 metastasis, most commonly involving the liver (20/31, 64.5%), lung (15/31, 48.4%), peritoneum (5/31, 16.1%), and bone (4/31, 12.9%). Most patients (23/31, 74.2%) experienced prior surgery. Over half (18/31, 58.1%) of the patients had an ECOG PS of 0.

**Table 1:** Baseline characteristics.

Characteristics	All patients (n=31)
Sex	
Male	23 (74.2%)
Female	8 (25.8%)
Age, years (range)	57 (51-66)
Primary tumor sites	
Colon	13 (41.9%)
Rectal	18 (58.1%)
Left colonic carcinoma	27 (87.1%)
Right colonic carcinoma	4 (12.9%)
Mutations	
*RAS/BRAF* wild-type	9 (29.0%)
Any mutation	12 (38.7%)
Unknown	10 (32.3%)
MSI/MMR status	
MSS/pMMR	11 (35.5%)
MSI-H/dMMR	0
Unknown	20 (64.5%)
Metastasis	
Liver	20 (64.5%)
Lung	15 (48.4%)
Peritoneum	5 (16.1%)
Bone	4 (12.9%)
Pelvic cavity	3 (9.7%)
Lymph nodes	3 (9.7%)
Adrenal gland	2 (6.5%)
Numbers of metastases	
1	3 (9.7%)
≥2	28 (90.3%)
Prior chemotherapy	
Yes	13 (41.9%)
Adjuvant chemotherapy	10 (32.3%)
Neoadjuvant chemotherapy	3 (9.6%)
No	18 (58.1%)
Prior surgery	
Yes	23 (74.2%)
No	8 (25.8%)
ECOG PS	
0	18 (58.1%)
1	13 (41.9%)

Data are median (IQR) or n (%).

ECOG, Eastern Cooperative Oncology Group Performance Status.

### Drug treatment and compliance

Sixteen (51.6%) patients subsequently received maintenance therapy following initial therapy. At the data cut-off (June 30, 2023), all 31 patients have discontinued treatment. Common reasons for discontinuation were disease progression (16/31, 51.6%), loss to follow-up (6/31, 19.4%), TEAEs (5/31, 16.1%), and other therapies (4/31, 12.9%). The regimens with bevacizumab plus chemotherapy (22.6%), capecitabine (16.1%), or cetuximab in combination with chemotherapy (12.9%) were commonly used in out-group patients (**Additional File 1: Table S3** **)**.

The median duration of treatment for patients was 6.3 (range, 0.0-17.2) months, with a median treatment cycle of 8.0 (range, 0.0-24.0). A total of 13 (42%) patients had TEAEs that resulted in dose reduction, interruptions, or delays, in which three (10%) patients due to TEAEs releated with anlotinib, and the others (90%) due to TEAEs releated with chemotherapy.

### Efficacy

**Figure 3:**
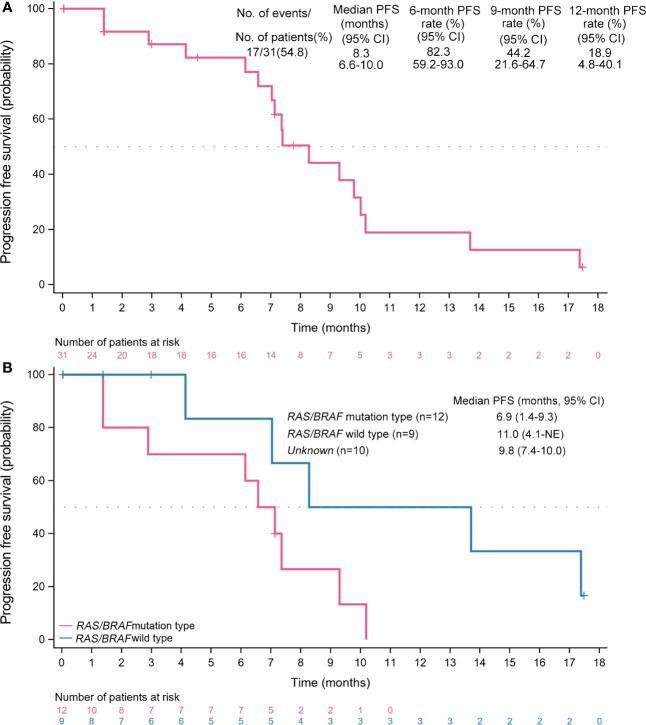
Kaplan-Meier analyses of survival. **(A)** Progression-free survival (full analysis set, n=31). **(B)** Progression-free survival across subgroups. PFS, progression-free survival; CI, confidence interval; NE, not evaluable.

The median follow-up time was 17.5 (95% CI, 3.0-17.5) months. Seventeen (54.8%) PFS events were observed in the FAS population. The median PFS was 8.3 (95% CI, 6.6-10.0; **Figure 3A**) months, with 6-, 9-, and 12-month PFS rates of 82.3% (95% CI, 59.2%-93.0%), 44.2% (95% CI, 21.6%-64.7%), and 18.9% (95% CI, 4.8%-40.1%), respectively. The PFS of maintenance therapy was also analyzed, which was named PFS2 and was defined as the time from maintenance therapy to disease progression or death from any cause. The median PFS2 was 4.1 (95% CI, 3.0-5.1) months in a total of 16 patients who received maintenance therapy. The median PFS was 11.0 (95% CI, 4.1-NE) months for mCRC with *RAS/BRAF* wild-type, and 6.9 (95% CI, 1.4-9.3) months for mCRC with mutation (**Figure 3B**).

**Figure 4:**
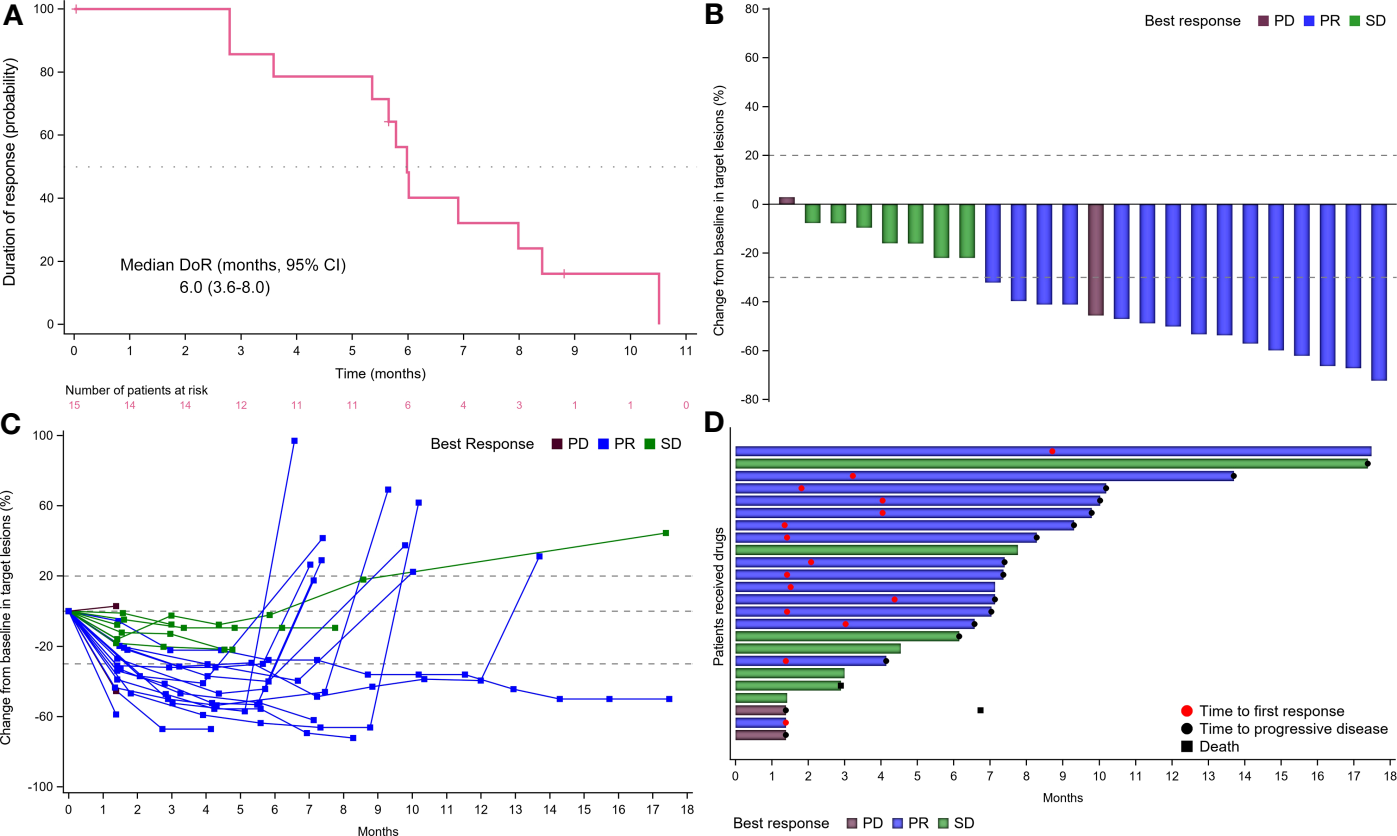
Tumor response. **(A)** Time to response and duration of response (DoR). **(B)** Waterfall plot of tumor size change from baseline to maximum percentage in each patient as per RECIST version 1.1. The blue column represented PR; the green column represented SD; the brown column represented PD. **(C)** Longitudinal change in tumor size from baseline. The blue line represented PR; the green line represented SD; the brown line represented PD. **(D)** Swimmer plots of the duration of treatment for patients with mCRC. The blue column represented PR; the green column represented SD; the brown column represented PD. CR, complete response; PR, partial response; PD, progressive disease; SD, stable disease; mCRC, metastatic colorectal cancer; RECIST, Response Evaluation Criteria in Solid Tumors.

No CR was observed from the FAS population and 15 (48.4%) patients achieved PR. Thus, the ORR was 48.4% (95% CI, 30.2%-66.9%) per RECIST version 1.1 (**Table 2**). Additional 7 (22.6%) patients had an SD, for a DCR of 71.0% (95% CI, 52.0%-85.8%). The ORR for patients with *RAS/BRAF* wild-type and any mutation was repectively 66.7% (95% CI, 29.9%-92.5%) and 60.0% (95% CI, 26.2%-87.8%). Of 15 responders, the median DoR was 6.0 (95% CI, 3.6-8.0; **Figure 4A**) months.

**Table 2:** Anti-tumor activity of anlotinib plus XELOX regimen.

Best responses	All patients (n=31)
PR	15 (48.4%)
SD	7 (22.6%)
PD	2 (6.5%)
NE	7 (22.6%)
ORR	15 (48.4%, 30.2%-66.9%)
DCR	22 (71.0%, 52.0%-85.8%)
Best responses	PPS population (n=24)
PR	15 (62.5%)
SD	7 (29.2%)
PD	2 (6.5%)
ORR	15 (62.5%, 40.6%-81.2%)
DCR	22 (91.7%, 73.0%-99.0%)

Data are n (%) or n (%, 95% confidence interval).

PR, partial response; SD, stable disease; PD, progressive disease; NE, not evaluable; ORR, overall response rate; DCR, disease control rate.

Moreover, of the 24 patients with evaluable imaging in the PPS, the ORR, and DCR were 62.5% (95% CI, 40.6%-81.2%) and 91.7% (95% CI, 73.0%-99.0%), respectively. Twenty-three (74.2%) obtained tumor shrinkage and 16 (51.6%) achieved decreased target lesion size (≤30%) from baseline (**Figures 4B, C**). Notably, despite decreased target lesion size of <30%, 1 patient was considered PD due to new lesions. One patient had the longest ongoing response of 17.3 months and the treatment duration for all participants was shown in **Figure 4D**.

### Safety and tolerability

All TEAEs of any grade with an incidence of >3% in the safety population were presented in **Table 3**. All 31 patients in the SAS experienced at least one TEAE, mainly including vomiting (58.1%), nausea (51.6%), decreased white blood cell count (51.6%), decreased neutrophil count (35.5%), hypertension (35.5%), diarrhea (25.8%), and peripheral neurotoxicity (22.6%). The majority were grade 1-2 TEAEs. Grade 3 or higher TEAEs occurred in 14 (45.2%) patients and primarily included decreased neutrophil count (12.9%), hypertension (12.9%), increased lipase (9.7%), hypertriglyceridemia (6.5%), and decreased lymphocytes count (6.5%). Besides, grade 3 or more oxaliplatin-related allergic reaction was reported in 1 (3.2%) of patients. Only 1 (3.2%) patient experienced grade 5 TEAEs (respiratory failure and heart failure). Four (12.9%) patients developed SAEs including abdominal pain, incomplete intestinal obstruction, anaphylaxis, bone marrow suppression (grade 4), and stroke. SAEs were considered irrelevant to the study drug. Dose reduction of anlotinib occurred in 5 (16.1%) patients due to TEAEs. Besides, 6 (19.4%) patients required chemotherapeutic dose reduction.

**Table 3:** All treatment-emergent adverse events (TEAEs) with an incidence of >3% in the SAS.

	Safety population (n=31)		
	Any grade	Grade 1-2	Grade 3 or more
Vomiting	18 (58.1%)	17 (54.8)	1 (3.2%)
Nausea	16 (51.6%)	16 (51.6%)	0
Decreased white blood cell count	16 (51.6%)	15 (48.4%)	1 (3.2%)
Decreased neutrophil count	11 (35.5%)	7 (22.6%)	4 (12.9%)
Hypertension	11 (35.5%)	7 (22.6%)	4 (12.9%)
Diarrhea	8 (25.8%)	7 (22.6%)	1 (3.2%)
Peripheral neurotoxicity	7 (22.6%)	7 (22.6%)	0
Decreased platelet count	6 (19.4%)	5 (16.1%)	1 (3.2%)
Hypokalemia	6 (19.4%)	6 (19.4%)	0
Hand-foot syndrome	5 (16.1%)	4 (12.9%)	1 (3.2%)
Hypoalbuminemia	5 (16.1%)	5(16.1%)	0
Fatigue	5 (16.1%)	5 (16.1%)	0
Astriction	5 (16.1%)	5 (16.1%)	0
Increased lipase	4 (12.9%)	1 (3.2%)	3 (9.7%)
Hypertriglyceridemia	4 (12.9%)	2 (6.5%)	2 (6.5%)
Proteinuria	4 (12.9%)	4 (12.9%)	0
Hypercholesterolemia	4 (12.9%)	4 (12.9%)	0
Hepatic insufficiency	4 (12.9%)	4 (12.9%)	0
Anemia	3 (9.7%)	2 (6.5%)	1 (3.2%)
Decreased lymphocytes count	3 (9.7%)	1 (3.2%)	2 (6.5%)
Positive fecal occult blood	3 (9.7%)	3 (9.7%)	0
Oxaliplatin-related allergic reaction	2 (6.4%)	1 (3.2%)	1 (3.2%)

Data are n (%).

## Discussion

The management of patients with mCRC was challenging given the aggressive nature of the disease and the limited choice of effective anti-tumor drugs (24). Although this study did not meet its primary endpoint of improving PFS, the moderate efficacy (mPFS, 8.3 months) may preliminarily suggest the anti-tumor activity of first-line anlotinib plus XELOX in mCRC.

In light of the efficacy analysis, the median PFS reported in the NO16966 trial regarding bevacizumab plus XELOX as the first-line therapy for mCRC was about 9 months, with an ORR of 47.0% (25, 26). In contrast, the present study demonstrated an unimproved median PFS of 8.3 months and a comparable ORR of 48.4%. It might be ascribed to the poor baseline status in our study over that in the NO16966 trial, such as ≥2 of metastases (90.3% vs. 62%) and perioperative chemotherapy (41.9% vs. 22.0%) (26). Moreover, the proportion of patients without efficacy evaluation due to loss to follow-up was up to 22.5% in this study. Of note, the previous C-002 study reported a slightly stronger survival and response compared to the results presented in our study (PFS, 11.3 vs. 8.3 months; ORR, 76.7% vs. 48.4%) (27). This finding was not unexpected due to a higher initial dose (12 vs. 10 mg) of anlotinib in the C-002 study, which may be considered more beneficial. Besides, all enrolled patients with *RAS/BRAF* wild-type mCRC might be important factors to achieve additional benefits in the C-002 study. It was also supported by numerically comparable median PFS (11.0 months) in our mCRC subpopulation with *RAS/BRAF* wild-type. Of note, the addition of anlotinib to the chemotherapy in the C-001 study may also contribute to better PFS, as evidenced by the moderate PFS (6.6 months) regarding chemotherapy alone as induction therapy in the OPTIMOX-2 study (23). Notwithstanding all patients with advanced-stage metastases, 71.0% of patients still had disease control in our study. It might reasonably assume that anlotinib may inhibit angiogenesis more comprehensively owing to its characteristics of multiple targets, as well as its ability to against chemotherapy resistance (28, 29).

Maintenance therapy, as a therapeutic strategy with less toxicity and stable efficacy, was essential for most mCRC patients following the initial therapy, which significantly reduced AEs and improved patients’ quality of life (QoL). Several clinical trials and meta-analyses on maintenance therapy have demonstrated favorable efficacy with EGFR inhibitors/bevacizumab with or without chemotherapy (30–32); however, the best maintenance scheme that could guide the selection of candidates, regimens, timing, and balance clinical benefits and costs have not been developed. Despite moderate survival benefits with PFS2 of 4.1 months in this study, one patient achieved an ongoing response of up to 17.3 months, which suggested that anlotinib alone as maintenance therapy may harbor potential benefits for mCRC patients who benefited from the initial therapy. In addition, palmar-plantar erythrodysesthesia syndrome, as the frequent TEAE related to chemotherapy (33), was not observed in our study. The potential reason was that anlotinib alone as maintenance therapy avoided the risk. However, acknowledging the limited sample size in this study, there remains no concrete evidence that anlotinib or not may intensify the toxicities of capecitabine or vice versa. Furthermore, no new safety signals or unmanageable safety profiles leading to death during the maintenance stage occurred. Taken together, these data importantly dictated that anlotinib alone might be considered a novel, suitable, and potential maintenance therapy meriting testing for this population compared with capecitabine plus anlotinib reported in the previous study (27).

It is critical to consider TEAEs when patients receive potentially effective drug combination regimens (34). Grade 1-2 TEAEs were more frequently reported in our study. Common TEAEs, such as nausea and vomiting, were usually tolerable and manageable and disappeared rapidly after symptomatic treatment. Overall, no unexpected safety profiles using this combination were observed compared with the C-002 study (27). The primary TEAEs of anlotinib identified in the previous review (15), including hypertension and proteinuria, were observed in this study. Intriguingly, these two TEAEs were more mild, tolerable, and manageable in our study over the C-002 study (hypertension: 35.5% vs. 86.7%; proteinuria: 9.7% vs. 10%) (27), which might be ascribed to the decreased dose of anlotinib in our study. In addition, the incidence of ≥grade 3 TEAEs in our study was lower than that in the NO16966 study evaluating XELOX alone (67.7% vs. 72.0%) (20). It might suggest no signs of increased toxicity despite the addition of anlotinib. Taken together, anlotinib plus XELOX followed by anlotinib monotherapy may be considered a potential routine treatment option for appropriate patients.

Taken together, the results reported in ALTER-C-001 and previous C-002 all indicated the feasibility of first-line anlotinib combined with XELOX for mCRC, especially for *RAS/BRAF* wild-type mCRC. Currently, standard first-line therapy with bevacizumab plus chemotherapy conferred superior survival benefits (8, 35, 36) and was available for *RAS/BRAF* wild-type mCRC in clinical practice. Considering the encouraging efficacy and limited choice of anti-angiogenic approaches, non-inferiority studies as compared to bevacizumab-based regimens are warranted for potentially active treatment options. A randomized, phase III study (NCT04854668) is in progress to prospectively compare the efficacy and safety of first-line CAPEOX plus bevacizumab or anlotinib in patients with *RAS/BRAF* wild-type mCRC (37). In parallel, a randomized non-inferiority trial involving *RAS/BRAF* status and primary tumor sites to evaluate the addition of anlotinib vs. bevacizumab to XELOX/FOLFOX is also required in the future.

Limitations of the present study included the single-arm design, the relatively limited sample size, and the absence of an OS analysis. Besides, this non-global study with data from multiple centers was only conducted in China, which might affect the generalizability of the results to a broader population. Overall, the present study still confirmed that anlotinib plus XELOX may be a promising first-line therapy for mCRC. However, further evaluation of this combination regimen in a randomized phase III trial with a larger sample size is warranted in the near future.

## Conclusions

Although this regimen failed to reach improvement of PFS as we expected, preliminary efficacy and manageable safety profiles indicated that some patients may achieve benefits with first-line anlotinib plus XELOX. Besides, our findings could also provide a framework for additional insight into the application of anlotinib monotherapy as a maintenance medication for mCRC patients who have benefited from the initial therapy. Importantly, larger-scale randomized clinical trials are required to further verify the feasibility of this regimen.

## Ethics approval and consent to participate

This study was approved by the Ethics Committee of Sichuan Cancer Hospital (No. SCCHEC-02-2019-018). Written informed consent was obtained from each patient. All procedures in the present study conformed to the ethical standards of the institutional and/or national research committee, Good Clinical Practice guidelines, and the 1964 Helsinki Declaration and its later amendments or comparable ethical standards.

## Data availability statement

The original contributions presented in the study are included in the article/**Supplementary Material**, further inquiries can be directed to the corresponding authors.

## Ethics statement

The studies involving humans were approved by Each center had independent ethics committee that approved the research protocol. This study was approved by the Ethics Committee of Sichuan Cancer Hospital (No. SCCHEC-02-2019-018). The studies were conducted in accordance with the local legislation and institutional requirements. The participants provided their written informed consent to participate in this study.

## Author contributions

Conception/Design: JY; Provision of study material or patients: BS, Y-WH, HH, S-JY, Y-DJ, C-LS, and JZ; Collection and/or assembly of data: HH, JZ, KZ, and BY; Data analysis and interpretation: BS, JZ, Y-WH, KZ, and BY; Manuscript writing: BS, HH, LZ, S-JY, Y-DJ, C-LS, JZ, HS, KZ, BY, Y-WH, and JY; Final approval of manuscript: BS, HH, LZ, S-JY, Y-DJ, C-LS, JZ, HS, KZ, BY, Y-WH, and JY.
