# Supplementary material for: Efficacy and safety of anlotinib plus XELOX regimen as first-line therapy for mCRC: a single-arm, multicenter, phase II study (ALTER-C-001)
Source: Front Oncol. 2023 Sep 1;13:1238553. doi: 10.3389/fonc.2023.1238553 (PMC10505961; doi:10.3389/fonc.2023.1238553)
Supplement: Supplementary file 1 [file DataSheet_1.docx]

**Supplementary tables**

**Table S1. Detailed exclusion criteria**

| Exclusion criteria |
| --- |
| Mucinous adenocarcinoma or ovarian implantation metastasis |
| Prior therapy of antiangiogenic agents |
| Give priority to surgery per investigator  High risk of bleeding or perforation due to the tumor’s obvious invasion of the adjacent organs or the presence of a formed fistula |
| Other malignancies within 5 years (other than cured cervical carcinoma in situ, non-melanoma skin cancer, and superficial bladder tumor) |
| Previous unrelieved treatment-related toxicity (> grade 1) per the NCI CTCAE v4.03 (excluding alopecia and nerve toxicity [≤ grade 2] induced by oxaliplatin) |
| Factors affecting oral medication (swallowing difficulty, chronic diarrhea, et al.) |
| Treatment of anti-tumor modern Chinese medicine preparation and immunomodulators^1^ approved by CFDA within 4 weeks prior to the study, or other Chinese medicine within 2 weeks before the study |
| Respiratory syndrome (respiratory dysfunction [≥ grade 2]) due to pleural effusion or ascites |
| Interstitial disease |
| Any severe and/or uncontrolled disease |
| Poor blood pressure control (systolic blood pressure ≥ 150 mmHg or diastolic blood pressure ≥ 100 mmHg) with one antihypertensive drug or good blood pressure control with ≥ 2 antihypertensive drugs |
| Myocardial ischemia, myocardial infarction, or arrhythmia (including QTc ≥ 480 ms) of > class II, congestive heart failure in New York Heart Association (NYHA) functional class II-IV |
| Active or uncontrolled severe infection (≥ grade 2) |
| Liver cirrhosis, decompensated liver disease, active or chronic hepatitis (hepatitis viral load > 1000 copies/ml) requiring antiviral treatment |
| Kidney failure requiring hemodialysis or peritoneal dialysis |
| History of immune deficiency (including HIV-positive, other acquired and congenital immunodeficiency diseases, or previous organ transplantation) |
| Poor diabetes control (fasting blood glucose [FBG] > 10 mmol/L) |
| Urinary protein ≥ ++, and confirmed 24-hour urinary protein > 1.0 g |
| Coagulopathy (INR > 1.5, prothrombin time [PT] > ULN+4 s, or APTT > 1.5 ULN) with bleeding tendency, thrombolytic or anticoagulant therapy^2^ |
| Epilepsy requiring treatment |
| Gastrointestinal disease with a bleeding tendency (eg. active peptic ulcer); or disease may result in gastrointestinal bleeding, perforation, or obstruction per investigators |
| Fatal haemorrhage because tumors currently or potentially invade the peripheral blood vessels per investigators |
| The major surgical operation, incision biopsy, or obvious traumatic injury within 28 days prior to study grouping |
| Tendency or history of bleeding diathesis; bleeding (≥ grade 3), non-healing wounds, ulceration, or fracture within 4 weeks prior to study grouping |
| Arterial and venous thrombosis within 6 months (eg. stroke [including tias], deep venous thrombosis, and pulmonary embolism) |
| A history of psychotropic substance abuse with the inability to quit, or dysphrenia |
| Prior or current brain metastases |
| Other anti-tumor trials within 4 weeks prior to the study |
| Concomitant diseases seriously endangering patients’ safety or interfering with the completion of the study |

^1^ Immunomodulators included lentinan, compound sophora, cinobufagin, kanglaite, panaxan, xiaoaiping, shenqifuzheng injection, bruceolic oil emulsion, kang-ai, thymosin, interferon, interleukin-2, bacillus calmette-guerin, transfer factor, levamisole, etc.

^2^ Note: Low-dose heparin (adult dose: 0.6-1.2×10^4^ U/day) or aspirin (dose: 100 mg/day) is permitted for prophylaxis if the international normalized ratio (INR) of prothrombin time is ≤ 1.5.

**Table S2. Detailed dose titration and delayed dose criteria**

| **Treatment-related adverse events** | **Administration time** | **Dose titration** |
| --- | --- | --- |
| Grade 0-2 | On-time delivery/Delay delivery | Normal dose |
| Grade 3 | Delay delivery till TRAEs<grade 2 | Reduce dose |
| Grade 4 | Delay delivery till TRAEs<grade 2 | Reduce dose or discontinue drug per investigators |
| Abnormal liver function (increased ALT, AST, or total bilirubin)  Grade 1  Grade 2 (normal baseline)  Grade 2 (abnormal baseline)  Grade 3  Grade 4  Proteinuria  Grade 1 (urine protein < 1.0 g [24 h])  Grade 2 (1.0 g ≤ urine protein < 2.0 g [24 h])  Grade 2 (2.0 g ≤ urine protein < 3.5 g [24 h])  Grade 3 (urine protein ≥ 3.5 g [24 h])  Decreased platelet count  Grade 1  Grade 2  Grade 3  Grade 4  Bleeding  Grade 1  Grade 2  ≥ Grade 3 | On-time delivery  Delay delivery till TRAEs<grade 2  On-time delivery  Delay delivery till TRAEs<grade 2  Discontinue the treatment  On-time delivery  On-time delivery  Delay delivery till TRAEs<grade 2 within 2 weeks  Delay delivery till TRAEs<grade 2 within 2 weeks  Delay delivery  Delay delivery  Delay delivery  Discontinue the treatment  On-time delivery  Delay delivery till TRAEs<grade 2 within 2 weeks  Discontinue the treatment | Normal dose  Reduce dose  Normal dose  Reduce dose  Discontinue the treatment  Normal dose  Normal dose  Reduce the dose or discontinue the treatment > twice  Reduce the dose or discontinue the treatment > twice  Normal dose till platelet count returned to baseline or normal values within 1 week  Normal dose till platelet count returned to baseline or normal values within 1 week; reduce dose if platelet count returned to baseline or normal values over 1 week  Reduce dose if platelet count returned to baseline or normal values within 2 weeks  Discontinue the treatment  Normal dose  Reduce dose  Discontinue the treatment |

**Table S3. Subsequent treatments in out-group patients.**

| **Treatment regimens** | **Patients** |
| --- | --- |
| Bevacizumab + chemotherapy (FOLFIRI/XELOX/CAPIRI) | 7 (22.6%) |
| Capecitabine | 5 (16.1%) |
| Cetuximab + chemotherapy (FOLFIRI) | 4 (12.9%) |
| Capecitabine + radiotherapy | 2 (6.5%) |
| FOLFIRI chemotherapy | 1 (3.2%) |
| Microwave/radiofrequency ablation for liver disease | 1 (3.2%) |
| Palliative treatment | 1 (3.2%) |
| Unknown | 9 (29.0%) |

Abbreviations: FOLFIRI=fluorouracil + irinotecan, XELOX=oxaliplatin + capecitabine, CAPIRI=capecitabine + irinotecan.
